# Volunteer Foreign Fighters in the Ukrainian Conflict and Considerations for Forensic Psychiatry: Toward an Interdisciplinary Dialogue

**DOI:** 10.3389/fpsyt.2022.914369

**Published:** 2022-05-19

**Authors:** Alexander Smith, Anna Buadze, Anish Ranjan Dube, Roman Schleifer, Michael Liebrenz

**Affiliations:** ^1^Department of Forensic Psychiatry, University of Bern, Bern, Switzerland; ^2^Department of Psychiatry, Psychotherapy and Psychosomatics, Psychiatric University Hospital Zurich, Zurich, Switzerland; ^3^Charles R. Drew University of Medicine and Science, Psychiatry Residency Program Faculty, Los Angeles, CA, United States

**Keywords:** Ukraine, foreign fighters, mental health, forensic psychiatry, risk assessment, expert testimony, advocacy

## Introduction

The current hostilities in Ukraine have seen a largescale influx of foreign fighters, with reports that both Russia and Ukraine are actively incorporating overseas nationals into their war efforts. This paradigm is reminiscent of past conflicts, such as the Spanish Civil War (1936–1939) and more recently the Islamic State (2014-present). Moreover, the new wave of recruitment extends previous deployments of foreign fighters to the region during tensions in Donbass that began in 2014. Amidst the ongoing crisis, the Russian President, Vladimir Putin, has welcomed the enrolment of foreign volunteers for Russia and expressly encouraged Syrian combatants to join (1). For their part, Ukrainian officials cited nearly 20,000 individuals signing up to the International Legion of Defense of Ukraine (2).

This opinion article focuses on those citizens who through their own initiative choose to deploy to the war in Ukraine from their country of domicile for non-financial reasons, as distinct from private military contractors and mercenaries; this is in line with comparable lexicon from the political sciences (3). Previous studies observed that motivations of foreign fighters were complex, variable, and contingent on the framework of individual wars. For example, foreign fighters journeyed to fight for and against the Islamic State out of kinship, religiosity, and political beliefs (4, 5). However, the mental health and mental state of prospective or returning foreign fighters, especially within the Ukrainian context, is a neglected topic in academic literature and wider press coverage.

We illustrate several potential practical and ethical issues of this phenomenon at the interface between psychiatry and the law. This paper is not intended to be exhaustive, but instead discusses the role of forensic expertise for civil- and criminal courts, such as in conducting risk assessments, determining therapeutic measures, and assessing for social security given the unique circumstances of these foreign fighters. As the war intensifies and burgeoning numbers travel to Ukraine, foreign fighters pose a multifaceted, cross-disciplinary, and increasingly immediate concern for our discipline.

## Foreign Fighters and Mental Health

Investigations into the mental state of prospective or returning foreign fighters are scarce, with several limitations, and are mainly restricted to the Islamic State, as highlighted by Dawson (6). Research tends to be descriptive, presumably because of data paucity and the difficulty of accessing these individuals for psychiatric assessments. Nonetheless, within the extant literature, there are suggestions that a proportion of foreign fighters might experience psychopathological symptoms and/or mental health disorders. For instance, Weenink examined a sample of Dutch foreign fighters traveling to Syria and identified several morbidities, including psychotic disorders, substance use disorders, autism spectrum disorder, attention deficit hyperactivity disorders, and posttraumatic stress disorder (7). Further, the Institute for Strategic Dialogue found that individuals with military backgrounds who journeyed to fight against the Islamic State cited psychological issues as part of their decision (8). In a Ukrainian context, Weijenberg and van Zuydewijn noted the presence of mental health disorders in case studies in Donbass (9), and there are corresponding indications for prospective foreign fighters during the current crisis (10).

Research has broadly illustrated the mental health burden of warfare (11, 12). Facing similar conditions, foreign fighters in Ukraine may well experience critical life events and witness death, threatened death, or become exposed to actual or threatened serious injuries. Such situations could be even more acute given contemporaneous accounts of Russian atrocities (13). All these factors could lead to the development or recurrence of psychiatric disorders.

Those traveling to Ukraine possess varying degrees of prior military involvement, from former veterans to individuals with no combat experience (14, 15). This is noteworthy as in a study of 289,328 American veterans from the wars in Iraq and Afghanistan, 36.9% received mental health diagnoses (16). Returning to a live combat zone may exacerbate existing psychiatric morbidities (17); an unfamiliar environment like Ukraine, combined with less immediate stress reduction strategies, could further accentuate these issues (18). In addition, for those without prior combat experience, first-time exposure to warfare might lead to both short- and long-term mental health disorders (19).

Isolation from relatives and support networks, insufficient knowledge of local languages, and inadequacies in wartime medical provisions are just some issues that may render foreign fighters susceptible to psychopathology (20). Additionally, deficiencies within Ukrainian psychiatric systems were evident even pre-conflict and there have been calls for the mental health needs of civilian combatants to be urgently addressed (21). Widespread availability of firearms may only destabilize an already alarming scenario, as might the prospect of foreign volunteers being thrust into the line of fire as “cannon fodder” (22).

Further, Russia has declared that it considers foreign fighters on the Ukrainian side to be “mercenaries” (23). Consequently, should they be taken captive, foreign fighters would be ineligible for protection or medical care under the Geneva Convention, and one can only speculate about their welfare; accounts of mistreatment are already emerging (24). Accordingly, we urge all parties in the conflict to safeguard the human rights and mental health needs of this population.

## Considerations for Forensic Psychiatry and Law

A proportion of foreign fighters who return to their home countries may require care for new or recurring mental health disorders that have not been sufficiently treated in the conflict zone. The substantial expertise in our discipline means that forensic psychiatrists can contribute to interdisciplinary information exchanges about foreign fighters. Below, we discuss a list of important considerations for forensic psychiatry and legal fields.

### Provision of Care in Pre-trial Detention

There are compound factors that could affect the provision of psychiatric care for foreign fighters. For instance, it is feasible that these individuals will return home under the threat of criminal consequences for not having initially reported their stay in the warzone and thus only belatedly seek psychiatric help.

Additionally, as governments become more sensitized, foreign fighters may encounter law enforcement agencies immediately on re-entry and be taken into custody. In different jurisdictions worldwide, it is the role of a forensic psychiatrist to provide clinical interventions in pre-trial settings. As has been noted, such care can be notoriously challenging and remains underdeveloped in many contexts (25, 26). Given complex legal processes and difficulties in gathering evidence, foreign fighters may face longer periods of pre-trial detention longer than offenders with conventional charges. This could thereby entail more extensive clinical care, especially as suicidality has been found to be prevalent amongst pre-trial inmates (27).

### Assessment for Executive Function Disorders

Owing to the high prevalence of neurodevelopmental disorders in forensic settings, there is extensive scientific and clinical expertise in the community concerning the impairments associated with deficits in executive functioning. Forensic psychiatrists and psychologists are therefore in a unique position to assess the role of impulsivity and rash-decision making, which are themselves key attributions of executive function disorders, in individuals choosing to join the conflict or its aftermath. We can help inform treatment procedures, healthcare policy, and requisite rehabilitation plans when foreign fighters return home (28).

### Advocacy

There has been prominent international uncertainty regarding the legal status of foreign fighters because of heterogeneous jurisdictional systems worldwide. The U.S. has discouraged civilians from volunteering to fight, but to the authors' knowledge, has issued no advice as to whether they would violate the Neutrality Act of 1794 (albeit unlikely) (29). The U.K. Foreign Secretary attested that she would support people who volunteered as foreign fighters, but UK officials have warned about future sanctions (30). Switzerland, which legally prohibits its nationals from participating in overseas conflicts, has said those who do so may face prosecution and risk losing their statehood (31). Other countries have issued analogous warnings to prospective foreign fighters (32). In our view, governments must clarify any ambivalence in legislation and be clear in their messaging, perhaps underlining how citizens can make a positive local contribution, for example through charities or official organizations. This is essential to protect the welfare of their populations and prevent vulnerable people from deploying to a foreign conflict.

### Extremism in Forensic Evaluations and Risk Assessments

Those who do volunteer to fight could pose a credible security threat and may require forensic psychiatric interventions. Political extremism has been highlighted in foreign fighters in other contexts such as in Syria (33), and as noted in the ongoing crisis, individuals who travel may have diverse ideologies (34). Since 2015, Rekawek from the Counter Extremism Project interviewed foreign fighters on both sides of the conflict in Ukraine and identified political extremism and its attendant security threat (35). Mackenzie and Kaunert reached similar conclusions, and like Rekawek, underlined this as an underreported concern (36). Whilst the extent of such beliefs for foreign fighters in the current hostilities are unknown and we do not seek to legitimize Kremlin propaganda by overstating their influence, their suggested presence may hold implications for the immediate security of civil society in Ukraine and might increase the risk of violence for some foreign fighters upon their return. Extremist ideology could coalesce with unfettered access to weaponry in the currently active war zone and therefore may constitute a plausible clinical concern for forensic psychiatrists (37). As a discipline, we must remain vigilant of the link between extremism and mental illness (38) when conducting forensic evaluations and risk assessments within this constituency.

### Expert Testimony

Forensic psychiatrists could be asked to provide expert evaluations of foreign fighters in legal proceedings. Criminal responsibility, intent, and premeditation are conceptually problematic given the composite framework of the Ukrainian crisis; motives will inevitably vary and may obfuscate objective diagnoses. Significantly, the consequences of these cases could be more severe than a typical criminal trial if they entail the loss of someone's statehood. Moreover, contemporaneous press coverage has largely conveyed a sympathetic view of foreign fighters who have honorable sentiments, are defending democracy, or are engaging in a selfless act by fighting for Ukraine. The presence of such a narrative in the public eye, as perpetuated by the media, may potentially leave forensic psychiatrists susceptible to biases in their decision-making, including the anchoring effect and confirmation bias (39). We must acknowledge these issues and uphold the best standards of practice for our patients and our profession.

Forensic psychiatrists should consider comprehensive forms of therapy, including evidence-based psychosocial and cognitive behavioral interventions in the treatment of veterans (40, 41), when advising courts on mandatory in- or outpatient treatments.

Moral and ethical questions arising from the societal duty of care for these foreign fighters may also have implications for our discipline. As a hypothetical example: if an individual returns from the war in Ukraine and cites a mental health issue incurred during this conflict as a causal factor in a disability benefit claim, how should we approach this situation? Reports suggest that analogous social debates may already be underway (10).

In such a scenario, state actors, private corporations, or even the media might seek to pressure or influence decisions that fall firmly within the realm of medicine. Forensic psychiatrists should remain vigilant for dual-agency bias.

Research has outlined the complexities of providing forensic expertise for social security claims amongst civilian and veteran populations (42, 43). Similar concerns would also be relevant during forensic evaluations in other jurisdictions and circumstances, for instance in child custody cases and firearm ownership applications. Increased training, education, and awareness programs for forensic psychiatrists could mitigate against such issues.

## Concluding Remarks

Foreign fighters deploying to Ukraine raise practical and ethical concerns for forensic psychiatry and the law. Foreign fighters with untreated mental health disorders upon return call for the specialized expertise of forensic psychiatrists. The unique characteristics of this constituency, coupled with the wider complexities of the Ukrainian conflict, makes this a problematic paradigm for our field. This opinion paper seeks to raise awareness about these issues rather than serve as a definitive guide. Ultimately, as a discipline, we must ensure that if a foreign fighter transitions from a battlefield in Ukraine to the courtroom or into a forensic setting, we afford them the best standard of practice and care.

## Author Contributions

AS, AB, RS, and ML conceptualized this paper. AS and ML wrote the first draft of the paper. AS, AB, AD, RS, and ML revised and edited sections of this opinion paper. All authors contributed to manuscript revision, read, and approved the submitted version.

## Funding

Open access funding provided by University Of Bern.

## Conflict of Interest

AB was born in Georgia and experienced the conflict of the Russo-Georgian War in 2008. RS was born in Ukraine and members of his family are currently caught up in the violence. The remaining authors declare that the research was conducted in the absence of any commercial or financial relationships that could be construed as a potential conflict of interest.

## Publisher's Note

All claims expressed in this article are solely those of the authors and do not necessarily represent those of their affiliated organizations, or those of the publisher, the editors and the reviewers. Any product that may be evaluated in this article, or claim that may be made by its manufacturer, is not guaranteed or endorsed by the publisher.
